# POCUS for Detection of Esophageal and Gastric Foreign Bodies

**DOI:** 10.24908/pocusj.v10i02.19230

**Published:** 2025-11-17

**Authors:** Daniella Lamour, Jacqueline Jean-Gilles, David Kinas, Edward Lopez, Robert A. Farrow

**Affiliations:** 1 Mount Sinai Medical Center, Miami Beach, FL, USA; 2 St. George's University School of Medicine, University Centre Grenada, West Indies, Grenada, GRD

**Keywords:** Esophageal foreign body, POCUS foreign body, Pediatric foreign body, POCUS upper GI tract, Point of care ultrasound

## Abstract

**Background:**

Foreign body ingestion is a common and challenging complaint for physicians in the emergency department (ED). Although most foreign bodies pass naturally without surgical intervention, about 10-20% require removal based on their size, shape, and location within the gastrointestinal tract. X-rays and computed tomography (CT) scans are frequently used for evaluation. However, x-rays cannot detect radiolucent foreign bodies, both modalities emit ionizing radiation, and CT scans are notably expensive. Point of care ultrasound (POCUS) offers a valuable alternative for detecting foreign bodies, as it avoids radiation exposure, reduces costs, and is readily accessible.

**Case Series:**

We present a case series involving three distinct instances of foreign body ingestion, where POCUS enhanced patient outcomes and disposition. This series includes evidence on use of POCUS for an obstructed esophageal foreign body and two gastric foreign bodies in a stable patient and unstable altered patient suspected of medication overdose.

**Conclusion:**

POCUS is an effective tool for identifying foreign bodies in the upper gastrointestinal tract, especially when x-rays fail to visualize non-radiopaque materials or when attempting to minimize radiation from CT scans. It allows emergency physicians to quickly confirm the presence and precise location of foreign bodies in the gastrointestinal tract, leading to faster clinical decision making and reduced risk of complications, such as perforation or obstruction.

## Introduction

Foreign body ingestion is a common and challenging complaint for physicians in the emergency department (ED), affecting both pediatric and adult populations. Children aged 6 months to 3 years are most vulnerable, while adults at risk include body packers and those with esophageal dysmotility or mental health disorders [1]. Various objects pose a significant threat to the gastrointestinal mucosa layer, including magnets, button batteries, and sharp items, all of which heighten the risk of perforation. Approximately 80% of ingested foreign bodies traverse the gastrointestinal tract without incident, however, the remaining 20% may become obstructed and require emergent removal [2]. X-rays and computed tomography (CT) scans are conventional imaging modes but point of care ultrasound (POCUS) should also be considered.

POCUS is a non-invasive, radiation-free imaging modality that can be interpreted at the bedside, making it a safe alternative for detecting foreign bodies [3]. Buonsenso et al. (2021) demonstrated that POCUS, despite being performed by pediatricians with minimal ultrasound training, achieved 100% accuracy in detecting ingested foreign bodies in the eight pediatric cases studied, underscoring its diagnostic reliability and ease of implementation [4]. We present a case series involving three distinct instances of foreign body ingestion, where POCUS enhanced patient outcomes and disposition. This series includes two pediatric patients who ingested radiolucent bottle caps—one obstructed in the esophagus and another in the stomach—as well as one unstable and altered adult patient suspected of medication overdose.

## Case 1

**Figure 1.**
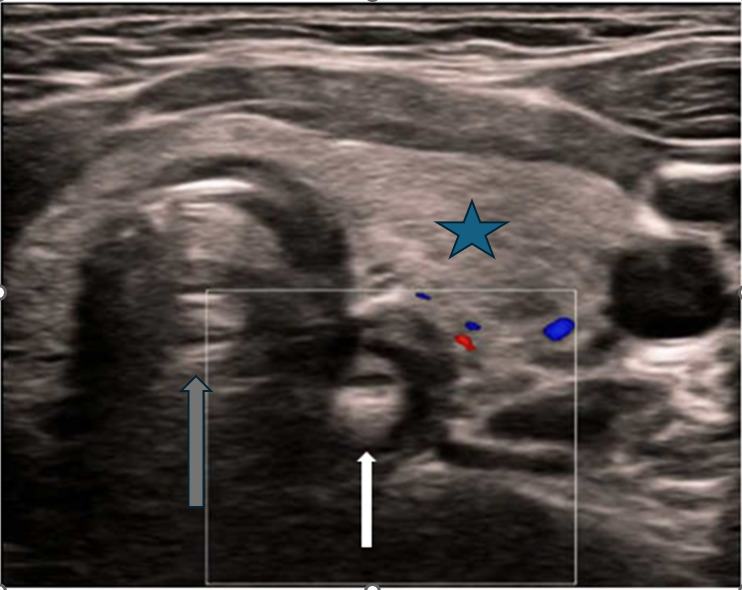
The white arrow highlights the foreign body, clearly identified by its marked hyperechoic signal within the anechoic lumen of the esophagus. The gray arrow indicates the trachea, located to the left of the esophagus. The blue star marks the overlying thyroid gland, visualized as a homogeneous, hyperechoic structure anterior to the trachea and esophagus.

**Figure 2.**
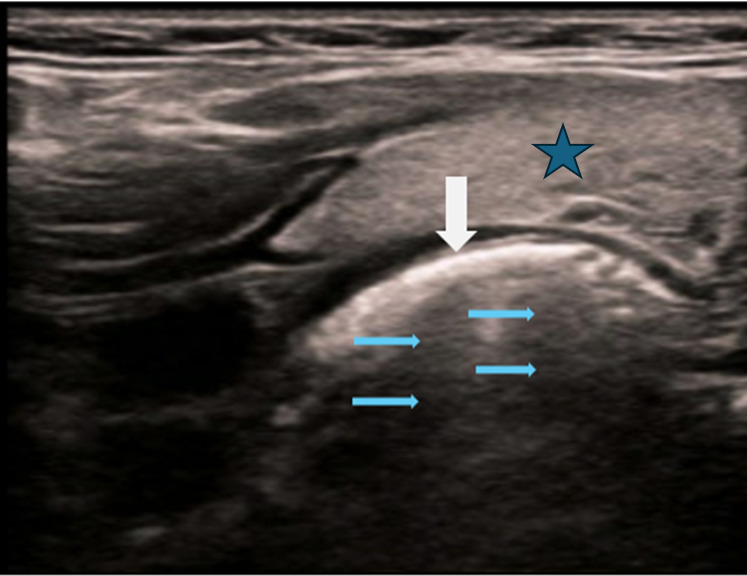
The foreign body (white arrow) is seen within the anechoic lumen of the cervical esophagus, accompanied by posterior acoustic shadowing (blue arrows). The blue star marks the overlying homogenous thyroid gland.

A healthy 11-year-old boy presented to the ED after swallowing a plastic bottle cap. The incident was witnessed by his mother who performed abdominal thrusts due to presumed choking. He subsequently complained of throat discomfort which improved by ED evaluation. Despite improved symptoms and negative radiography, there was still heightened suspicion of a foreign body ingestion, thus POCUS evaluation of the esophagus was performed. A high-frequency linear probe was placed in the transverse plane of the anterior neck to locate the cervical esophagus, located just to the right of the trachea—a large, round, anechoic structure with hyperechoic tracheal rings. A hyperechoic object was identified within the esophagus in the transverse plane (Figure 1). The high-frequency linear probe was then positioned in the longitudinal plane along the anterior aspect of the neck to isolate and visualize the cervical esophagus. In this orientation, the trachea was no longer visible, allowing the esophagus to be the primary focus, while the overlying thyroid gland appeared as homogeneous, hyperechoic tissue. Figure 2 displays the same foreign body (white arrow) within the anechoic lumen of the cervical esophagus, accompanied by posterior acoustic shadowing (blue arrows). POCUS confirmed an obstructing esophageal foreign body, prompting consultation with gastroenterology and the patient's transfer to a pediatric facility for emergent removal. A bottle cap was successfully extracted, leading to a rapid and uncomplicated recovery.

## Case 2

**Figure 3.**
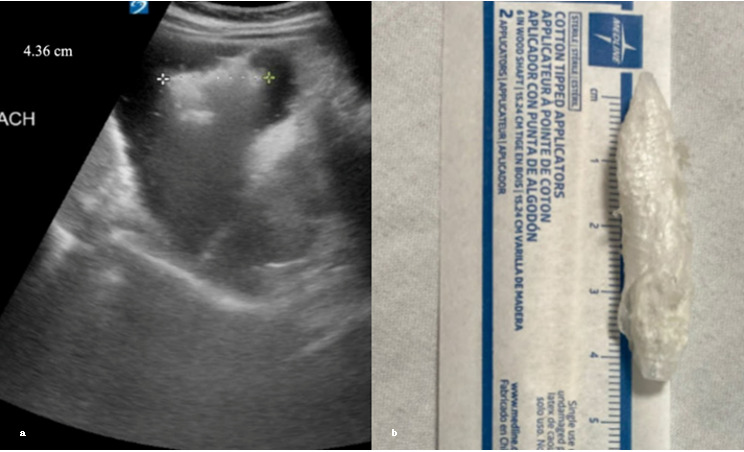
Figure 3a reveals a hyperechoic object with irregular and jagged margins within the anechoic lumen of the stomach, measuring 4.36 cm. Figure 3b displays an identical bottle cap that the patient was chewing on, also measuring 4.36 cm.

A healthy 8-year-old boy presented with his mother with concern of possibly swallowing a plastic bottle cap. The ingestion was unwitnessed, making it uncertain whether the patient had swallowed anything; therefore, a gastric POCUS evaluation was performed to investigate further. POCUS was initially inconclusive due to lack of gastric dilation and mixed contents. We asked the patient to drink water, which further distended the stomach while providing a better medium for visualization. He also assumed a left lateral decubitus position to shift the bowel gas away. With the patient lying on his left side, a curvilinear probe was placed in the transverse plane over the epigastric area and we scanned the region until the stomach was identified. POCUS revealed a hyperechoic object with irregular and jagged margins within the anechoic lumen of the stomach, measuring 4.36 cm (Figure 3a). Notable dirty shadowing and reverberation artifacts were present, consistent with the acoustic properties of plastic. In Figure 3b, the patient's mother displayed an identical bottle cap—approximately 4.3 cm in size—that her son had been chewing, confirming foreign body ingestion. With this confirmation, the pediatric gastroenterologist advised outpatient surgery follow-up within 1-2 weeks and strict return precautions. During a follow-up call, the mother confirmed that the patient expelled the bottle cap within seven days without experiencing any complications, indicating a complete and uneventful recovery.

## Case 3

**Figure 4.**
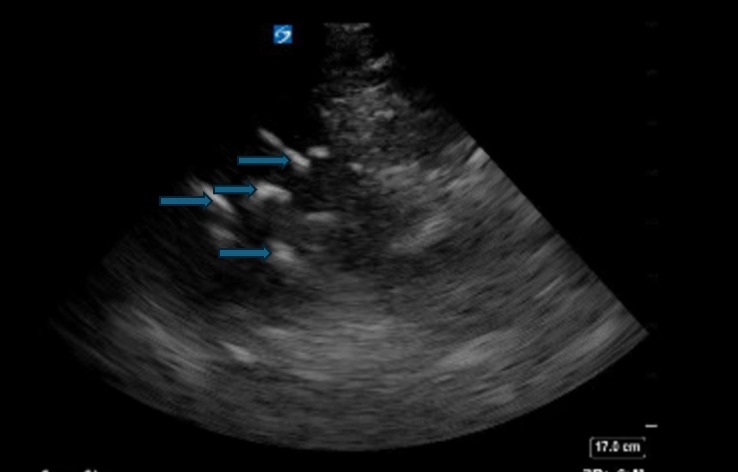
POCUS image displaying multiple hyperechoic objects (blue arrows) within the stomach lumen.

A 30-year-old woman was brought to the ED by emergency medical services after being found unresponsive at home with unmarked empty medication bottles and a suicide note. She was intubated on arrival for hypoxia and an unresponsive state. There were no signs of trauma, and her pupils were reactive and equal. Chest x-ray and abdominal x-ray were negative other than slight pneumonitis. Gastric POCUS was performed using a phased array probe placed in the epigastric region. It revealed several well-defined hyperechoic structures in the stomach lumen, seen in Figure 4, reinforcing the suspicion of an acute ingestion. Her electrocardiogram had prolonged QRS and QTc intervals. Prior to admission to the intensive care unit, these clinical factors led to the administration of sodium bicarbonate, activated charcoal, and whole bowel irrigation, as advised by the poison control center. The patient required an extended stay in the intensive care unit due to the severity of her condition but was ultimately stabilized and discharged home with psychiatric support.

## Discussion

Diagnosis of foreign bodies in the upper gastrointestinal tract is heavily dependent on patient history, examination, and imaging. It can still prove challenging, particularly in vulnerable populations. POCUS is a valuable imaging modality in these cases. Most foreign bodies will display a leading hyperechoic edge relative to surrounding tissue and an element of posterior reverberation artifacts or acoustic shadowing, depending on the object's composition and surface characteristics [5]. Additionally, flat and highly reflective materials such as glass, metal, or wood with retained air will produce reverberation artifact posteriorly, while objects with irregular surfaces and small curvature produce anechoic shadowing [6]. Surface characteristics of the foreign bodies' macro echotexture can further aid in identifying the object. Given the potential for esophageal obstruction and intestinal perforation, prompt identification and risk stratification for expedited removal is imperative [7].

Pediatric patients have increased vulnerability to foreign body ingestions yet are often one of the most difficult patient populations to get a detailed history and exam. In another case series, POCUS effectively detected foreign bodies in the stomach and esophagus, even in cases where traditional radiographic methods failed to identify the object, emphasizing the ability to provide a rapid and accurate diagnosis in pediatric emergency settings [8]. In the first case, a young male presented after a witnessed ingestion. Despite the improvement in symptoms, the possibility of a missed esophageal foreign body remained. The vast majority of esophageal foreign bodies lodge in the thoracic inlet, as with our patient, which is the most common site of obstruction due to its anatomical narrowing and physiological constriction [9]. On POCUS, the cervical esophagus, which is usually collapsed, appears in the left paratracheal space [10]. In our case, this exhibited an inner hyperechoic structure with the macro echotexture of a bottle cap. With heightened clinical suspicion combined with POCUS confirmation, we urgently transferred him for pediatric gastroenterology intervention and the bottle cap was successfully removed.

In our second case, gastric ultrasound following water ingestion and patient repositioning revealed a 4.36 cm hyperechoic foreign body, later confirmed to be a chewed bottle cap. This observation supports findings by Yamamoto et al. (2017), who demonstrated that strategic positional changes can enhance gastric ultrasound by helping foreign bodies float into view—an easily implemented technique requiring minimal additional training [11]. Horowitz et al. 2016 reported that POCUS identified gastric foreign bodies in three pediatric cases [12]. Two of these were confirmed with standard radiographs, while the third, not identified radiographically, was passed in the feces. All three objects were initially found in the stomach using POCUS. These studies reinforce the value of POCUS as a reliable, noninvasive diagnostic tool. Our patient successfully passed the bottle cap without complications, requiring no additional imaging or interventions, demonstrating the usefulness of POCUS in safely guiding conservative treatment and avoiding unnecessary radiation exposure. Broader adoption among frontline providers could significantly reduce dependence on radiography, decrease pediatric radiation exposure, and facilitate faster, safer clinical decision-making.

While foreign body ingestions are more common in pediatrics, we present a third case in which gastric POCUS revealed multiple hyperechoic particles in an adult patient with suspected suicide attempt due to a drug overdose. Activated charcoal administration is typically not recommended after the first hour as absorption into the bloodstream may have already occurred [13]. Given the uncertainty surrounding the patient's ingestion time, POCUS confirmed the presence of pills in the stomach, suggesting an acute ingestion. In a study by Amitai et al. (1992) it was shown that ultrasound could detect pills in the stomach, particularly sustained-release or enteric-coated preparations [14]. This finding prompted the timely initiation of treatment as recommended by the poison control center, and patient did improve throughout her stay in the intensive care unit. Although more studies are needed to determine the accuracy of POCUS as a detection tool for pills in the stomach and its significance, it is an invaluable diagnostic tool. POCUS enhances clinical decision-making in gastrointestinal emergencies, particularly in vulnerable populations where rapid, noninvasive assessment is critical to guide timely and effective treatment.

## Conclusion

Foreign body ingestions are a common presenting complaint in the ED, with management strategies influenced by factors including the type of ingestion, the size and shape of the foreign body, presenting symptoms, and the location within the gastrointestinal tract. This case series strengthens the current literature by demonstrating the diagnostic breadth of POCUS for the identification of ingested foreign bodies in a variety of clinical settings, including the ingestion of esophageal and gastric bottle caps in pediatric patients and suspected pill overdoses in adults. It highlights POCUS as a rapid, noninvasive, and radiation-free imaging technique that can be effectively applied in pediatric and adult EDs, thereby improving diagnostic efficiency, guiding timely interventions, and improving patient outcomes.

## References

[1] Lee JH. Foreign body ingestion in children. Clin Endosc 2018;51(2):129–136. 10.5946/ce.2018.039 29618175 PMC5903088

[2] Dereci S, Koca T, Serdaroğlu F, Akçam M. Foreign body ingestion in children. Turk Pediatri Ars 2015;50(4):234–240. 10.5152/TurkPediatriArs.2015.3164 26884693 PMC4743866

[3] Niset A, Baert J, Dupriez F. Point-of-care ultrasound for the diagnosis of pediatric foreign body ingestion: A narrative review and illustrative case report. Pediatr Emerg Care 2023;39(9):728–733. 10.1097/PEC.0000000000002997 37339160

[4] Buonsenso D, Chiaretti A, Curatola A, Morello R, Giacalone M, Parri N. Pediatrician-performed point-of-care ultrasound for the detection of ingested foreign bodies: Case series and review of the literature. J Ultrasound 2021;24(1):107–114. 10.1007/s40477-020-00512-7 32212088 PMC7925727

[5] Marin JR, Abo AM, Rowell MA. Not all radiopaque foreign bodies shadow on ultrasound: Unexpected sonographic appearance of a radiopaque magnet. Pediatr Emerg Care 2014;30(11):796–798. 10.1097/PEC.0000000000000261 25364959

[6] Suramo I, Päivänsalo M, Vuoria P. Shadowing and reverberation artifacts in abdominal ultrasonography. Radiology 1985;155(1):191–196. 10.1148/radiology.155.1.3888630 3888630

[7] Ozkan Z, Kement M, Kargı AB, Censur Z, Gezen FC, Vural S, Oncel M. An interesting journey of an ingested needle: a case report and review of the literature on extra-abdominal migration of ingested foreign bodies. J Cardiothorac Surg 2011;26;6:77. doi: 10.1186/1749-8090-6-77 PMC3123183 21615959

[8] Salmon M, Doniger SJ. Ingested foreign bodies: A case series demonstrating a novel application of point-of-care ultrasonography in children. Pediatr Emerg Care 2013;29(7):870–873. 10.1097/PEC.0b013e31829e63d3 23823272

[9] Mori T, Nomura O, Hagiwara Y. Another useful application of point-of-care ultrasound: Detection of esophageal foreign bodies in pediatric patients. Pediatr Emerg Care 2019;35(2):154–156. 10.1097/PEC.0000000000001729 30702544

[10] Kawai Y, Ogawa O, Hirose Y. Point-of-Care Ultrasound for an Esophageal Foreign Body. J Emerg Med 2022;63(2):e53–e56. doi: 10.1016/j.jemermed.2022.04.035 35871027

[11] Yamamoto LG, Yada RT, Inaba AS, Yamamoto JA. Diagnosis of ingested foreign body in the stomach by point-of-care ultrasound in the upright and slightly forward tilting position (bowing position). Clin Pediatr Emerg Med 2017;18(2):128–132. 10.1016/j.cpem.2017.01.010 28376070

[12] Horowitz R, Cico SJ, Bailitz J. Point-of-care ultrasound: A new tool for the identification of gastric foreign bodies in children? J Emerg Med 2016;50(1):99–103. 10.1016/j.jemermed.2015.07.022 26409678

[13] Juurlink DN. Activated charcoal for acute overdose: A reappraisal. Br J Clin Pharmacol 2015;81(3):482–487. 10.1111/bcp.12793 26409027 PMC4767212

[14] Amitai Y, Mitchell AA, McGuigan MA, Lovejoy FH, Woolf AD. The use of ultrasound in the diagnosis of oral drug overdose. Clin Toxicol 1992;30(3):443–450. 10.3109/15563659209000342

